# Immunological Signatures after *Bordetella pertussis* Infection Demonstrate Importance of Pulmonary Innate Immune Cells

**DOI:** 10.1371/journal.pone.0164027

**Published:** 2016-10-06

**Authors:** René H. M. Raeven, Jolanda Brummelman, Larissa van der Maas, Wichard Tilstra, Jeroen L. A. Pennings, Wanda G. H. Han, Cécile A. C. M. van Els, Elly van Riet, Gideon F. A. Kersten, Bernard Metz

**Affiliations:** 1 Intravacc, Bilthoven, The Netherlands; 2 Division of Drug Delivery Technology, Leiden Academic Centre for Drug Research, Leiden, The Netherlands; 3 Centre for Infectious Disease Control, National Institute for Public Health and the Environment (RIVM), Bilthoven, The Netherlands; 4 Centre for Health Protection (GZB), National Institute for Public Health and the Environment (RIVM), Bilthoven, The Netherlands; Universidad Nacional de la Plata, ARGENTINA

## Abstract

Effective immunity against *Bordetella pertussis* is currently under discussion following the stacking evidence of pertussis resurgence in the vaccinated population. Natural immunity is more effective than vaccine-induced immunity indicating that knowledge on infection-induced responses may contribute to improve vaccination strategies. We applied a systems biology approach comprising microarray, flow cytometry and multiplex immunoassays to unravel the molecular and cellular signatures in unprotected mice and protected mice with infection-induced immunity, around a *B*. *pertussis* challenge. Pre-existing systemic memory Th1/Th17 cells, memory B-cells, and mucosal IgA specific for Ptx, Vag8, Fim2/3 were detected in the protected mice 56 days after an experimental infection. In addition, pre-existing high activity and reactivation of pulmonary innate cells such as alveolar macrophages, M-cells and goblet cells was detected. The pro-inflammatory responses in the lungs and serum, and neutrophil recruitment in the spleen upon an infectious challenge of unprotected mice were absent in protected mice. Instead, fast pulmonary immune responses in protected mice led to efficient bacterial clearance and harbored potential new gene markers that contribute to immunity against *B*. *pertussis*. These responses comprised of innate makers, such as *Clca3*, *Retlna*, *Glycam1*, *Gp2*, and *Umod*, next to adaptive markers, such as CCR6^+^ B-cells, CCR6^+^ Th17 cells and CXCR6^+^ T-cells as demonstrated by transcriptome analysis. In conclusion, besides effective Th1/Th17 and mucosal IgA responses, the primary infection-induced immunity benefits from activation of pulmonary resident innate immune cells, achieved by local pathogen-recognition. These molecular signatures of primary infection-induced immunity provided potential markers to improve vaccine-induced immunity against *B*. *pertussis*.

## Introduction

The resurgence of pertussis in the vaccinated population prompts the necessity of more knowledge on effective immunity against *Bordetella pertussis* [1, 2]. This global health problem occurs after vaccination with acellular pertussis vaccines (aPV) and whole-cell pertussis vaccines (wPV) as recent data point out [3]. aPV-vaccinated individuals face waning immunity early after vaccination, since the vaccine-induced immunity lasts for only 4–12 years [4, 5] despite multiple booster vaccinations [6]. Moreover, research in baboons revealed that aPV-vaccinated animals are protected against disease, but still harbor viable bacteria resulting in continuation of pathogen transmission [7]. These findings indicate that even aPV-vaccinated individuals may act as an important resource for the transmission of *B*. *pertussis* [8, 9]. Hence, the present situation necessitates the reevaluation of pertussis immunity and vaccination strategies.

The immunity induced by a *B*. *pertussi*s infection may provide crucial information for improving vaccine-induced immunity, since it provides important advantages compared to immunity induced by aPV or wPV. Infection-induced immunity continues for a longer period [4, 5] and it leads to sterilizing immunity in mice and baboons [7, 10]. Infection as well as aPV and wPV vaccination induces strong systemic responses, but infection induces a mucosal immune response at the site of pathogen entry in addition. As others and we recently showed, this immunity includes pulmonary IgA, systemic IgG [10] and specific Th1/Th17 responses [10–12]. The lungs are naturally exposed to pathogens and therefore equipped with an epithelial layer and a large number of immune cells. The lung epithelial cells offer a first line of defense by secretion of anti-microbial peptides and pathogen recognition, but also by interaction with the local innate immune cells [13]. These innate immune cells include mucin-secreting goblet cells [14], alveolar macrophages [15] and the upper layer of the bronchus-associated lymphoid tissue (BALT) [16], that act in the same way as microfold (M)-cells [17], harbor functions, such as pathogen recognition and antigen-uptake. These pulmonary innate cells orchestrate the specific adaptive immune responses, such as mucosal IgA production and activation of tissue-resident B-cells and T-cells in the BALT [16].

In this study, we further explored the molecular and cellular events underlying infection-induced protective immune responses. To this end, pulmonary transcriptomic profiles were characterized around the challenge of mice protected by primary infection-induced immunity, in comparison to events in unprotected counterparts. Additionally, pulmonary and systemic cytokine profiles as well as cellular and antibody mediated immune responses against *B*. *pertussis* were studied. Markers for pulmonary immunity included both trained innate and adaptive signatures. M-cells, alveolar macrophages and epithelial cells characterized the former, and CCR6^+^ B-cells, CCR6^+^ Th17 cells, CXCR6^+^ T-cells and mucosal IgA the latter. These extensive insights into the signatures of infection-induced immunity involving important pulmonary components may be used for the development of improved pertussis vaccines with long lasting immunity.

## Results

### Pre-Existing Immunological Signatures in Protected Mice before *B*. *pertussis* Challenge

**Fig 1:**
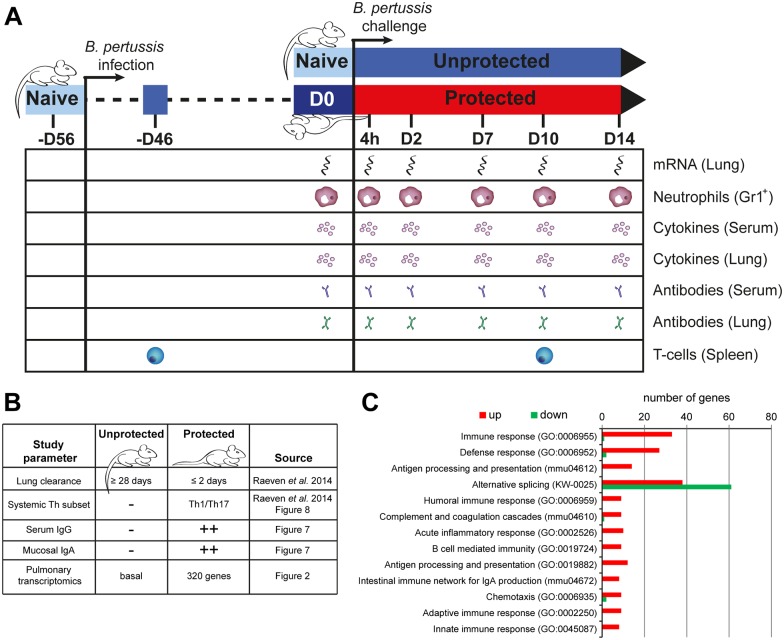
Design and baseline parameters of a *B*. *pertussis* challenge model in protected and naive unprotected mice. (A) Schematic diagram of animal pre-treatment, sacrifice, sampling and systems analysis on 4 hours and 2, 7, 10 and 14 days p.c. in a *B*. *pertussis* challenge model in protected and unprotected BALB/c mice. Pulmonary transcriptomic profile, percentage of splenic Gr1^+^ cells (neutrophils), serum and lung cytokine profiles, serum and lung antibody profiles, and specific splenic CD4^+^CD44^+^ T-cells were assessed at the given time points in protected and unprotected mice. (B) Study parameters at baseline (D0) of naive unprotected mice and protected mice, 56 days after primary infection, including time frames of lung clearance, systemic T-helper subsets, serum IgG profile, mucosal IgA, and pulmonary transcriptomic profile as obtained from data in the current study and data adapted from our previous study [10]. 320 genes are differential expressed in protected mice compared to naive (D0 unprotected) mice. (C) An overrepresentation analysis was performed using DAVID for Keywords, KEGG-pathways, and gene ontology biological pathways (GO-BP) to determine the function of the 320 genes. For each term, the number of upregulated (red) and downregulated (green) genes are depicted.

Recovery from a *B*. *pertussis* infection in BALB/c mice is associated with sterilizing immunity. A challenge with *B*. *pertussis* bacteria showed that the lungs were cleared within two days in these protected mice, whereas this takes approximately 28 days in unprotected mice [10]. To understand infection-induced protection in more detail we designed a systems biology approach to study pre- and post-challenge immune responses in unprotected and protected mice, recovered from a primary infection received 56 days before (Fig 1A). Before the receiving the challenge inoculum (D0), protected mice showed enhanced levels of *B*. *pertussis*-specific serum IgG, mucosal IgA in the lungs, and Th1/Th17 cells in the spleen, which were absent in naive (D0 unprotected) mice (Fig 1B). When comparing levels of pulmonary gene expression, 320 genes were still differentially expressed, mostly upregulated, in the lungs of protected compared to naive (D0 unprotected) mice (S1 Table). An overrepresentation analysis (ORA) was performed using DAVID [18] to ascribe the functional group to which the 320 corresponding proteins belong (Fig 1C). The data analysis indicated that the pulmonary gene expression of protected mice was enriched with 33 and 27 genes involved in immune response and defense response, respectively. Upregulated genes were further involved in antigen processing, the innate response and several terms indicating B-cell mediated immunity. Notably, a large number of both downregulated and upregulated genes were related to alternative splicing. In conclusion, these results indicated that the protected mice with infection-induced immunity contain a large variety of adaptive and additionally still some innate markers to resist a new *B*. *pertussis* challenge as compared to their unprotected naive counterparts.

### Pulmonary Transcriptome of Protected and Unprotected Mice Receiving a *B*. *pertussis* Challenge

**Fig 2:**
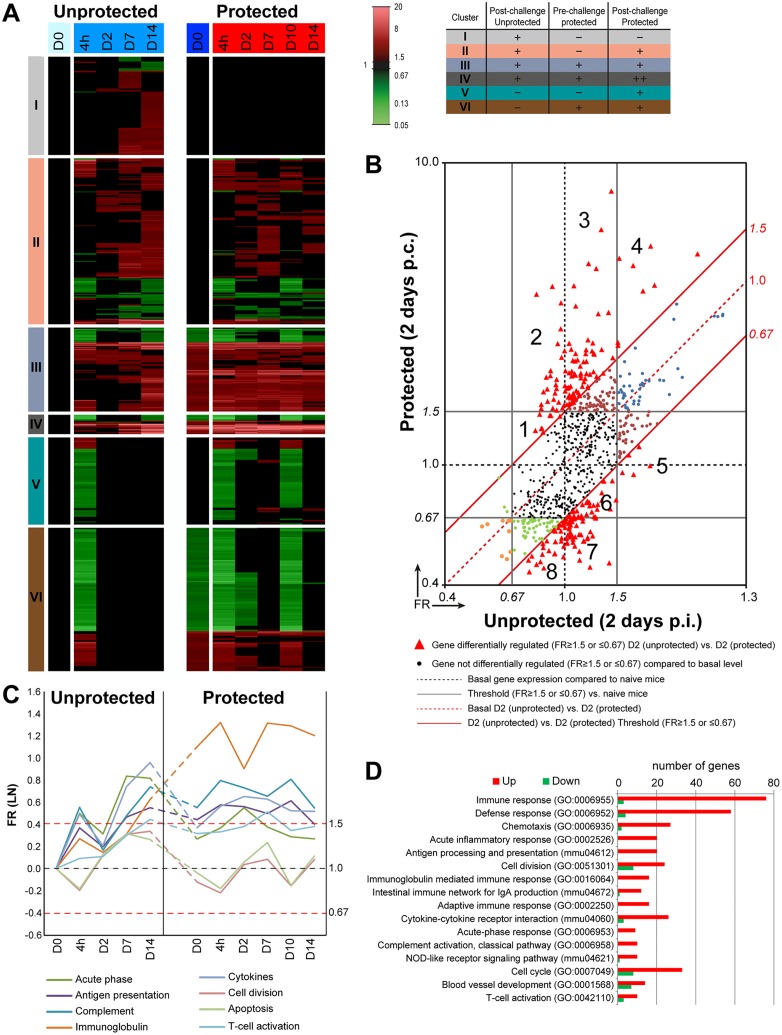
Pulmonary gene expression profiles in protected and unprotected mice following a *B*. *pertussis* challenge. (A) Fold changes in gene expression of both unprotected and protected mice were calculated compared to naive mice (D0 unprotected). The expression results (FR≥1.5, p-value≤0.001) are visualized as heatmap (mean of n = 3). Genes not exceeding a fold change of 1.5 are depicted as basal level (black) at this time point. In total, 786 genes were found to be differentially regulated. Genes were divided in six clusters (I-VI) based on their expression profiles (color coding for these clusters is depicted in an additional table): cluster I (Differential expression in unprotected mice, absent in protected mice), Cluster II (Differential expression in unprotected mice and protected mice), Cluster III (Differential expression in unprotected mice and in protected mice before and after challenge), Cluster IV (Differential expression in unprotected mice and additional differential expression as result of challenge in protected mice), Cluster V (Absent in unprotected mice but differential expression in protected mice) and Cluster VI (absent in unprotected mice but differential expression pre- and post-challenge in protected mice). (B) Transcriptomic profiles obtained on 2 days p.i. in unprotected and 2 days p.c. protected mice were compared by plotting all 786 genes in a scatter plot and divide the genes in different fractions based on co-expression. The black solid lines are the thresholds for the significant FR (FR ≥ 1.5 or ≤ 0.67) compared to naive mice (D0 unprotected) for both unprotected and protected mice. Black dots represent genes that are not significantly regulated compared to naive mice (D0 unprotected) in both groups. The red solid lines represent the threshold for the significant FR (FR ≥ 1.5 or ≤ 0.67) of both unprotected 2 days p.i. and protected mice 2 days p.c. All red triangles represent genes that show significant differential expression (FR ≥ 1.5 or ≤ 0.67) between unprotected 2 days p.i. and protected mice 2 days p.c. In total, 212 genes were differentially expressed between both groups of which 108 genes were upregulated and 104 were downregulated. These genes were divided in eight fractions that are significantly up-regulated (1–4) or downregulated (5–8) in protected mice compared to unprotected mice and are further specified as heatmaps in S1B Fig. Dots with other colors (orange, green, brown, and blue) represent genes that are significantly regulated in unprotected and/or protected mice compared to naive mice (D0 unprotected) but these genes are not differentially regulated between unprotected 2 days p.i. and protected mice 2 days p.c. (C-D) A selection of eight terms (KEGG-pathways and GO-BP terms) found enriched in the ORA of the 786 genes and the kinetics over time of indicated terms is depicted. (C) Kinetics was determined by averaging the FR for each term at each time point and is expressed on LN-scale. (D) For each enriched term, the Benjamini score and the number of upregulated (red) and downregulated (green) genes in the protected mice and unprotected mice is shown.

To gain more insight into gene transcription patterns associated with fast clearance of *B*. *pertussis* and protection, we subsequently assessed the pulmonary transcriptome over a period of 14 days post challenge (p.c.) in protected and post infection (p.i.) in unprotected mice in comparison to naive non-challenged mice (unprotected D0) (Fig 2A). In total, 786 genes were differentially regulated (*p*-value ≤ 0.001, fold ratio (FR) ≥ 1.5) (S1 Table). These genes were divided in six clusters (I—VI) based on differential expression in unprotected and protected mice (Fig 2). Cluster I: differential expression in unprotected mice, absent in protected mice; cluster II: differential expression in both unprotected mice and protected mice; cluster III: differential expression in unprotected mice and in protected mice pre- or post-challenge; cluster IV: differential expression in unprotected mice and additional differential expression post-challenge in protected mice; cluster V: absent in unprotected mice but differential expression in protected mice; cluster VI: absent in unprotected mice but differential expression pre- and post-challenge in protected mice.

The individual fold ratios (FR) of 786 differentially expressed genes were plotted for each time point after being analyzed in protected and unprotected mice (S1A Fig). Before the challenge (D0), already 320 genes were differentially regulated in the protected mice if compared to the unprotected mice. Furthermore, this analysis revealed that on 2 days p.c. the largest number of genes was differentially expressed between both groups, namely 212 genes of which 108 genes were upregulated and 104 were downregulated. Since the sterilizing immunity of protected mice resulted in *B*. *pertussis* clearance within 2 days p.c. [10], signatures underlying this immunity could be detected on 2 days p.c. in protected mice. Therefore, the transcriptomic profiles of the 786 genes were investigated in detail on 2 days p.c. (Fig 2B). Subsequent to the comparison of both datasets to naive mice, the fold ratios of each gene was calculated between the datasets of unprotected versus protected mice obtained 2 days p.c. (FR ≥ 1.5 or ≤ 0.67, red triangles) (Fig 2B). The scatter plot was divided in eight different fractions that were significantly upregulated (1–4) or downregulated (5–8) in protected mice compared to unprotected mice 2 days p.c. The genes of these fractions were shown as heatmaps (S1B Fig). Many genes are involved in innate (e.g. *A2m*, *Saa3*, *C3*, *Reg3g*, *Umod*) and adaptive (e.g. *Cd4*, *Ighg*, *Cxcr6*, *Ccr6*, *Cd22*) immune responses were upregulated in protected mice.

An ORA for KEGG pathways and GO-BP terms was performed on all 786 genes. In total, 97 terms were enriched (Benjamini ≤ 0.05). The number of upregulated and downregulated genes and the kinetics for a subset of these terms was determined (Fig 2C and 2D). Enriched terms contained genes involved in *immune response* (79 genes) including innate immune functions such as acute phase (9 genes), antigen presentation (20 genes), chemotaxis (29 genes), and pathogen recognition through the NOD-like receptor (11 genes). Moreover, terms incorporated in the adaptive immune response (16 genes) involved antibody response (16 genes), IgA responses (13 genes) and T-cell activation (13 genes) (Fig 2D). The expression kinetics were determined by averaging the FR for each time point for a selection of these enriched terms (Fig 2C). This analysis showed that post-challenge in protected mice the immunoglobulin-mediated immune response remained high (4h-D14). The complement response, antigen-presentation, and T-cell activation were still moderately enhanced in protected mice. Furthermore, genes belonging to acute phase, cytokine responses, apoptosis and cell division were expressed to a lesser extent upon challenge in protected mice than in unprotected mice.

While the ORA gave an insight in several functions involved upon challenge of protected as well as unprotected mice, a large number of genes that were differentially regulated in our dataset were not annotated. To obtain in depth information on the function of these genes, we performed additional text mining on all individual genes using BioGPS, GeneRifs, and the literature (Pubmed). Based on this information, selected genes could be grouped according to function such as T-cell-related responses, B-cell-related responses, membrane receptors and secreted proteins, as is described in more detail hereafter.

#### Gene expression of membrane receptors and secreted proteins

**Fig 3:**
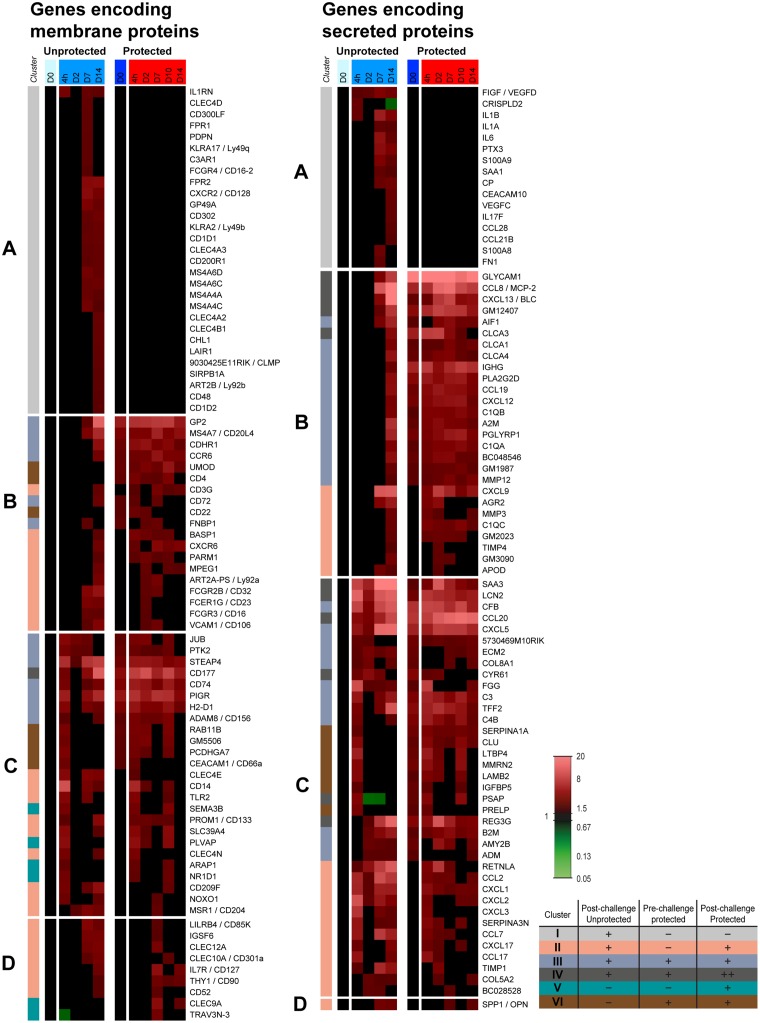
Pulmonary gene expression profiles of membrane proteins and secreted proteins in protected and unprotected mice following *B*. *pertussis* challenge. Expression profiles of genes in the lungs encoding membrane proteins (left panel) and secreted proteins (right panel) were selected according to GO-BP terms, KEGG pathways and text mining. Each list is divided in four groups (A-D) based on their expression profile on 4 hours and 2 days p.c. Group A contains genes that are significantly upregulated in unprotected mice, but unaltered in protected mice. Group B covers genes that are not upregulated on 4 hours and 2 days p.i. in unprotected mice, but are directly activated in protected mice. Group C comprehends genes that are upregulated on 4 hours and/or 2 days p.c. in both protected and unprotected mice. Group D includes genes that are significantly regulated from 7 days p.c. in both protected and unprotected mice. In addition, the color codes of the six clusters from Fig 2 are added.

The gene expression profiles of membrane receptors and secreted proteins indicated the involvement of specific cell types and biological processes. Therefore, the expression of all genes encoding for membrane proteins and secreted proteins was further investigated (Fig 3) and divided in four groups (Group A-D), based on their expression profiles in unprotected and protected mice during the first two days following challenge as follows:

Genes in group A were mainly upregulated in unprotected mice 7–14 days p.i., but unaltered in protected mice (Fig 3). Therefore, this gene expression is essential for a first encounter with *B*. *pertussis*, but it is not required for protected mice.

In group A, membrane marker genes were in general related to myeloid cells (*Cd300lf*, *Fcgr4*, *Gp49a*, *Cd200r1*, *Cd302*, *Lair1*, *Sirpb1a*). Other membrane marker genes are specific, for example for neutrophils (*Cxcr2*, *Fpr1*, *Fpr2*) or for macrophages, such as *Il1rn*, killer-cell lectin-like receptors (*Klra2*, *Klra17*), C-type lectins (*Clec4a2*, *Clec4a3*, *Clec4b1*, *Clec4d*) and membrane-spanning four-domains, subfamily A (*Ms4a6c*, *Ms4a6d*, *Ms4a4a*, *Ms4a4c*). Moreover, the expression of *Cd1d1* and *Cd1d2*, involved in MHC Class I antigen presentation, *C3ar1* (APCs, Macrophages, T-cell activation), *Clmp* (cell-cell adhesion molecule), and *Chl1* was only upregulated in unprotected mice. In addition, expression of some T-cell-related genes (*Art2b*, *Cd48*) were found. *Art2b* is constitutively expressed on T-cells, whereas *Cd48* is expressed on multiple immune cells playing a major role in T-cell activation. Finally, podoplanin (*Pdpn*) which is a lung injury marker and serves as a CD4^+^ effector T-cell inhibitor [19], was only expressed in unprotected mice. Among the genes encoding secreted proteins, expression of a number of acute inflammatory genes was altered in unprotected mice (*Cp*, *Fn1*, *Il1a*, *Il1b*, *Il6*, *Ptx3*, *Saa1*, *S100a9*, *S100a8*), but not in protected mice. Some genes are involved in blood vessel formation (*Fn1*, *Vegfc*, *Vegfd*) or LPS-binding (*Crispld2*). Furthermore, the genes of a Th17-specific cytokine (*Il17f*), and mucosal chemokines attracting IgA plasmablasts (*Ccl28*) and CCR7^+^ lymphocytes (*Ccl21b*) were only upregulated in unprotected mice.

The genes in group B were not expressed until 7 days p.i. in unprotected mice. However, in protected mice, these genes were already upregulated between 4 hours and 2 days p.c. (Fig 3). Here, higher activity of microfold cells (M-cells) was suggested by enhanced gene expression of *Gp2* and *Umod*. Moreover, macrophage-related genes (*Basp1*, *Mpeg1*, *Ms4a7*), innate-specific receptors such as Fc-receptors (*Fcgr2b*, *Fcer1g*, *Fcgr3*) and genes important for function of multiple innate cells (*Fnbp1*, *Parm1*, *Cdhr1*) were expressed earlier in protected mice. Additionally, gene expression of membrane markers (*Vcam1*, *Cd22*, *Cd72*, *Cd4*, *Cd3g*, *Cxcr6*, *Ccr6*, *Art2a-ps*) demonstrated earlier adhesion of immune cells in the lungs of protected mice. It indicated early presence of adaptive immune cells such as B-cells (*Cd22*, *Cd72*, *Ccr6*) and T-cells (*Cd4*, *Cd3g*, *Cxcr6*, *Ccr6*, *Art2a-ps*). In total, 16 genes encoding secreted proteins were still upregulated in protected mice before challenge, whereas seven genes were reactivated following the challenge. These genes included many chemoattractants (*Ccl8*, *Cxcl9*, *Cxcl12*, *Cxcl13*, *Ccl19*, *Glycam1*, *Gm12407*, *Gm2023*, *Gm1987*). Some genes are associated with attracting specific cells, such as B-cells (*Cxcl13*), CXCR4^+^ lymphocytes (*Cxcl12*), CCR7^+^ lymphocytes (*Ccl19*), CD62l^+^ lymphocytes (*Glycam1*), CXCR3^+^ T-cells (*Cxcl9*), and CCR5^+^ cells (*Ccl8*). In addition, upregulation was detected for genes associated with antibodies (*Ighg*), complement (*C1qa*, *C1qb*, *C1qc*), activated macrophages (*Aif1*), Peyer’s patches (*Pglyrp1*), matrix metalloproteinases (*Mmp3*, *Mmp12*) and an MMP inhibitor (*Timp4*), increased mucus production by goblet cells (*Agr2*, *Clca1*, *Clca3*, *Clca4*) and genes related to lipid metabolism (*Apod*, *Pla2g2d*). Expression of *Pla2g2d* in DCs and macrophages is related to induction of anti-inflammatory lipid mediators [20].

Most genes in group C were upregulated early (4 hours) after challenge in protected and unprotected mice (Fig 3). Analyzing the membrane markers, the expression of 12 genes was still upregulated prior to the challenge of protected mice, while the other 13 genes were reactivated post-challenge. The membrane marker genes in group C included a neutrophil specific marker (*Cd177*), for which the expression was earlier and more intense in protected mice. A similar observation was found for *Pigr*, which traffics IgA across the epithelial barrier. The C-type lectins (*Clec4e*, *Clec4n*), found on DCs, were detected in unprotected mice till 7 days p.i., but were only expressed in protected mice 4 hours p.c. The early expression of *Cd14* suggests LPS binding in both protected and unprotected mice, whereas *Tlr2* is involved in pathogen recognition. Additionally, markers for antigen presentation (*Cd74*, *H2-d1*), macrophages (*Msr1*), regulation of anti-inflammatory activities in murine airways (*Adam8*) [21], epithelial cells (*Pigr*, *Rab11b*, *Retnla*), DCs (*Cd209f*), and the endothelial marker *Plvap*, which enables lymphocytes to enter the lungs [22], were detected in this group of genes. Among the genes encoding secreted proteins, a selection of acute phase genes (*Saa3*, *Reg3g*, *Lcn2*) were highly upregulated in protected and unprotected mice. However, the expression went down after 2 days p.c. whereas this continued in unprotected mice. In protected mice, *Cxcl5* remained highly upregulated while *Cxcl1*, *Cxcl2*, and *Cxcl3* were expressed for a limited amount of time. The chemokines encoded by *Cxcl1*, *Cxcl2*, *Cxcl3* and *Cxcl5* are involved in CXCR2^+^ leukocyte recruitment. Expression of *Ccl20*, the chemokine attracting CCR6^+^ cells, was increased more intensely in protected mice than in unprotected mice. In addition, genes involved in the complement system (*Cfb*, *C3*, *C4b*), cytokine expression (*Ccl2*, *Ccl7*, *Ccl17*, *Cxcl17*), mucosal tissue repair (*Tff2*), epithelial cells (*Cyr61*), and mast cells (*5730469M10RIK / Fam213a*) were observed in both groups.

Genes in group D were mainly up-regulated in protected and unprotected mice between 7 and 14 days p.c. The expression of these genes returned faster to basal level in protected mice than in unprotected mice (Fig 3). Based on the membrane-related genes in group D, this phase indicates innate and adaptive immune cells interacting with each other (*Clec9a*, *Clec10a*, *Clec12a*, *Il7r*, *Thy1*, *Trav3n-3*). For example, genes were detected related to T-cells (*Il7r*, *Thy1*, *Trav3n-3*), DCs (*Clec9a*, *Clec10a*, *Clec12a*), leukocytes (*Lilrb4*), macrophages (*Igsf6*) and lymphocytes or myeloid cells (*Cd52*). Group D also contained the upregulated gene *Spp1*. The corresponding protein of *Spp1* is also known as osteopontin (OPN), which is produced by many different immune cells and possesses many immunological functions [23]. However, the expression of this gene was not different between protected and unprotected mice.

#### B-cell and antibody-related signatures

**Fig 4:**
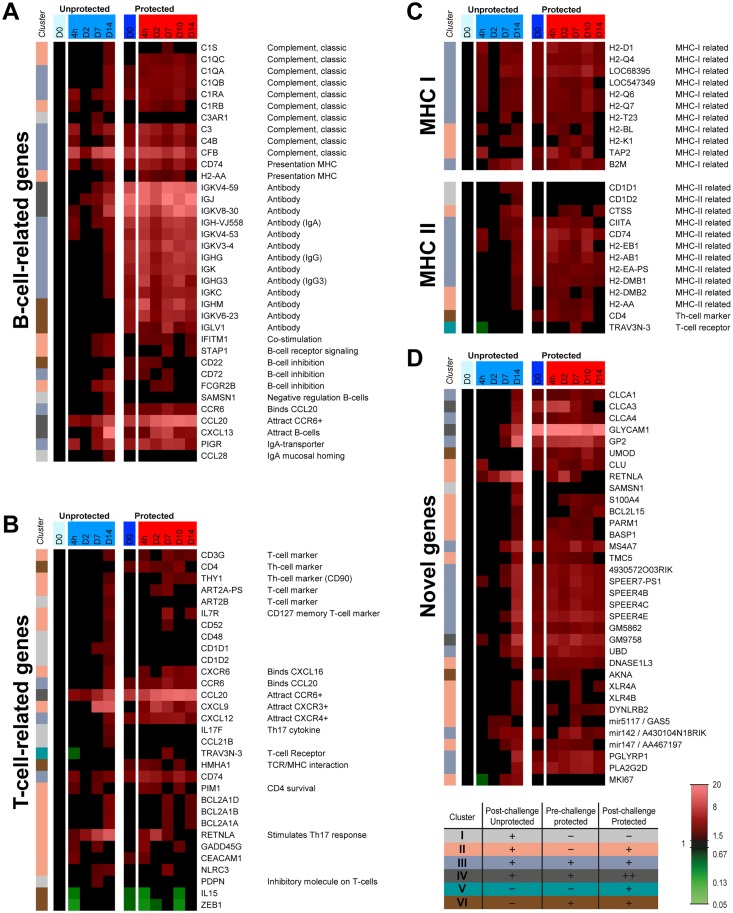
Pulmonary gene expression profiles of genes related to T-cells, B-cells, MHC-I and II and novel genes in protected and unprotected mice following *B*. *pertussis* challenge. Expression profiles of genes in the lungs related to (A) T-cells, (B) B-cells, (C) and antigen presentation by MHC-I and MHC-II were selected according to GO-BP terms, KEGG pathways and text mining. In addition, genes with unknown or poorly understood function that showed interesting gene expression profiles in protected mice were listed as (D) novel genes. Additionally, the color codes of the six clusters from Fig 2 are added.

The enhanced gene expression for the B-cell chemoattractant *Cxcl13*, detected 7 days p.i., suggested recruitment of B-cells towards the lungs of unprotected mice (Fig 4A). In protected mice, the expression of *Cxcl13* had raised earlier at 2 days p.c. Increased expression of antibody-related genes indicated B-cell recruitment to the lungs 14 days p.i. in unprotected mice. Higher and earlier expression of the immunoglobulin-related genes (i.e. *Igj*, *Igk*, *Ighm*, *Igkc*) was observed in protected mice than in unprotected mice (Fig 4A). These immunoglobulin-related genes indicated the formation of IgM, IgG and IgA by these B-cells in the lung. Notably, a small decrease in expression of antibody-related genes occurred 2 days p.c. in protected mice. Furthermore, genes of the classic complement pathway were co-expressed with the immunoglobulin-related genes. Genes of B-cell inhibitory receptors (*Cd22*, *Cd72*, *Fcgr2b*) were expressed slightly earlier in protected mice compared to unprotected mice. Moreover, the co-stimulatory receptor *Ifitm1* was earlier and more constant in protected mice. The upregulation of *Ccl28* in unprotected mice 14 days p.i. indicated homing of IgA producing B-cells to the mucosa. However, the expression of *Ccl28* was absent in protected mice. The upregulation of *Pigr*, the IgA transporter on epithelial cells, suggested an increase of IgA in the mucosa of protected and unprotected mice.

#### T-cell-related signatures

Gene expression of T-cell-related markers (*Cd3g*, *Cd4*) indicated the presence of T-cells, most likely T-helper (Th) cells, in the lungs of unprotected mice around 14 days p.i. (Fig 4B). The upregulation of *Cd3g* and *Cd4* is still present in protected mice prior to the challenge. The enhanced RNA levels remain high during challenge of these mice. In addition, multiple T-cell-related genes (*Il7r*, *Cxcr6*, *Thy1*) were upregulated 14 days p.i. in unprotected mice. The expression in protected mice was observed earlier at 7 days p.c., indicating a faster recall of memory T-cell responses. Expression of the IL-7 receptor gene (*Il7rI*) indicated the presence of memory T-cells. The Th17 specific cytokine gene (*Il17f*) was significantly upregulated in unprotected mice, but unaffected in protected mice. Resistin-like alpha (*Retnla*) was earlier expressed by epithelial cells in protected mice, and the expression was already diminished 7 days p.c. Resistin-like alpha stimulates Th17 responses [24].

#### Antigen presentation

Genes related to MHC class I and II were upregulated in unprotected mice following the challenge (Fig 4C). Whereas enhanced MHC-I expression was observed during the whole course of the challenge, MHC-II expression was found upregulated 7–14 days p.i. In protected mice, genes related to both antigen-presenting pathways were expressed at every time point and decreased 14 days p.c.

#### Genes not previously described in the context of immunity against *B*. *pertussis*

Some genes showed increased expression in response to *B*. *pertussis* challenge, but limited or no information is available about their function (Fig 4D). The expression of most of these genes was induced during the adaptive phase in unprotected mice and persisted in protected mice after challenge. Therefore, these genes may play an important role in immunity against *B*. *pertussis*. These genes include members of the SPEER-family (*4930572o03rik*, *Speer7-ps1*, *Speer4b*, *Speer4c*, *Speer4e*, *Gm5862*, *Gm9758*), chloride channel calcium activated channels (*Clca1*, *Clca3*, *Clca4*), microRNAs (*Mir142*, *Mir147*, *Mir5117*), a transcription factor (*Akna*), and X-linked lymphocyte-regulated genes (*Xlr4a*, *Xlr4b*). Significant expression of cell proliferation marker Ki-67 was found in unprotected mice, but unaffected in protected mice. Moreover, the expression of M-cell specific genes (*Gp2*, *Umod*), an effector protein of CD4^+^ CD25^+^ regulatory T-cells (*Pla2g2d*) [25], and of *Glycam1*, *Pglyrp1*, and *Retnla* were expressed earlier or to a stronger extent in protected mice compared to unprotected mice yet their involvement is not previously described in the context of immunity against *B*. *pertussis*.

### Modulated Cytokine Levels in Lung Tissue Extract

**Fig 5:**
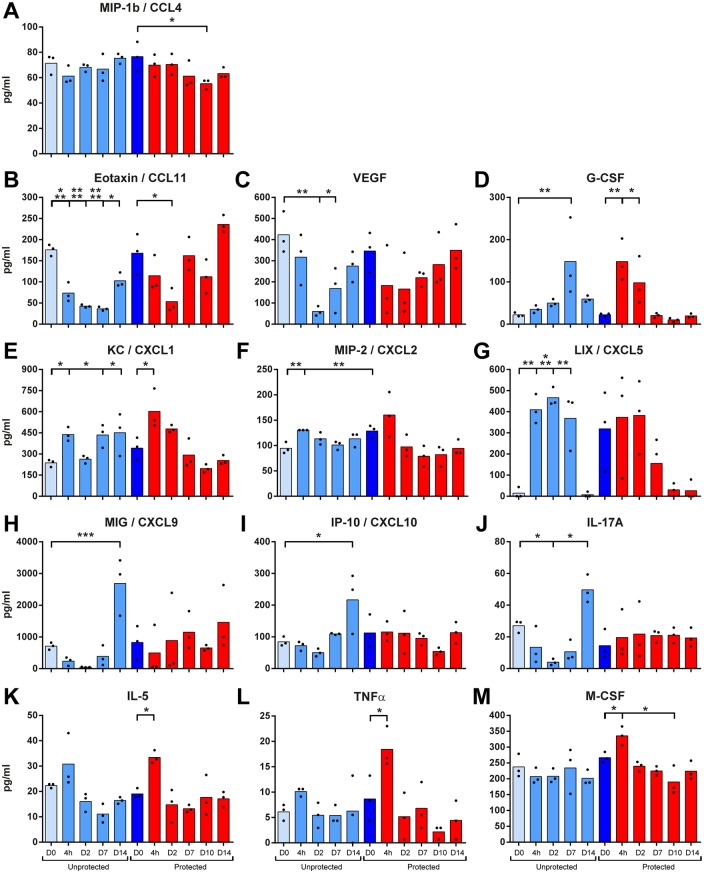
Cytokine profiles in the lungs following *B*. *pertussis* challenge in unprotected and protected mice. Pulmonary concentrations of (A) CCL4, (B) CCL11, (C) VEGF, (D) G-CSF, (E) CXCL1, (F) CXCL2, (G) CXCL5, (H) CXCL9, (I) CXCL10, (J) IL-17A, (K) IL-5, (L) TNFα and (M) M-CSF were analyzed before and after a *B*. *pertussis* challenge in unprotected (lighter blue bars) and protected (dark blue and red bars) mice, as indicated. Data represented as mean concentrations of individual values (n = 3). Significant values were calculated by one-way ANOVA with multiple comparison compared to the pre-challenge level (D0) of unprotected mice or protected mice (* = *p*<0.05, ** = *p*<0.01, and *** = *p*<0.001, **** = *p*<0.0001).

The protein concentrations of 32 cytokines in the lungs were analyzed. The concentrations of thirteen cytokines significantly changed following a *B*. *pertussis* challenge in protected and unprotected mice (Fig 5). CCL4 was decreased in protected mice 10 days p.c. (Fig 5A). CCL11 decreased in unprotected mice until 14 days p.i. and in protected mice until 2 days p.i. (Fig 5B). Moreover, a significant decrease occurred for VEGF in unprotected mice between 2–7 days p.i., which was less dramatic in protected mice (Fig 5C). In unprotected mice, G-CSF concentrations were elevated 7 days p.i. In protected mice, higher concentrations of G-CSF were found already at 4 hours and 2 days p.c. (Fig 5D). Both CXCL1 and CXCL2 were enhanced in unprotected mice, whereas only CXCL1 was significantly increased 4 hours p.c. in protected mice (Fig 5E and 5F). CXCL5 was significantly induced in unprotected mice and remained elevated until 7 days p.i. (Fig 5G). CXCL9, CXCL10 and IL-17A were elicited 14 days p.i. in unprotected mice, but they were absent in protected mice (Fig 5H–5J). IL-5, TNFα and M-CSF were produced in the lungs of protected mice 4 hours p.c., but not in unprotected mice (Fig 5K–5M).

### Enhanced Cytokine Levels in Mouse Serum

**Fig 6:**
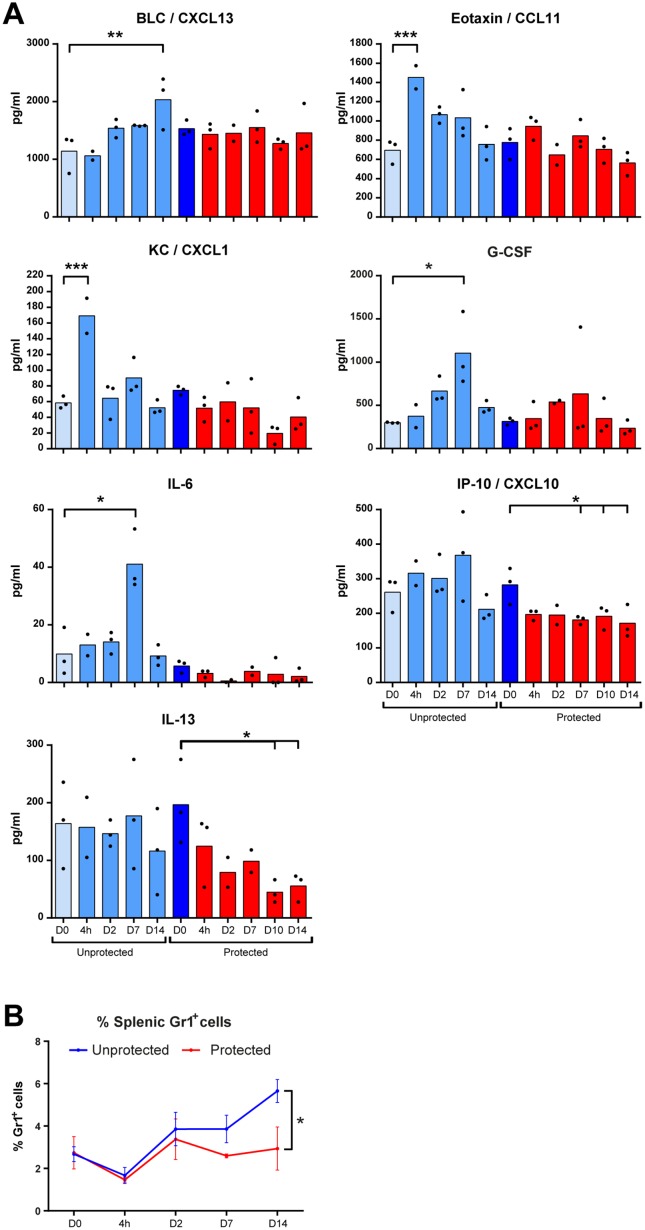
Serum cytokine profiles and percentage of splenic neutrophils following *B*. *pertussis* challenge in unprotected and protected mice. (A) The serum concentrations of 33 cytokines were analyzed before and after a *B*. *pertussis* challenge in unprotected (lighter blue bars) and protected (dark blue and red bars) mice, as indicated. Concentrations of CXCL13, CCL11, CXCL1, G-CSF, IL-6, CXCL10, and IL-13 serum were significantly altered and represented as mean concentrations of individual values (n = 3). Significant values were calculated by one-way ANOVA with multiple comparison compared to the pre-challenge level (D0) of unprotected mice or protected mice (* = *p*<0.05, ** = *p*<0.01, and *** = *p*<0.001). (B) The percentage of Gr1^+^ cells (neutrophils) was determined over time in the spleen of unprotected and protected mice by using Flow cytometry (* = *p*<0.05).

The concentrations of 33 cytokines were analyzed in serum of protected or unprotected mice before and following the *B*. *pertussis* challenge. Only for seven of these, significant alterations in their levels were found upon challenge (Fig 6A). The concentrations of CXCL1 (KC) and CCL11 (Eotaxin) were increased 4 hours p.i. in unprotected mice. Higher concentrations of both IL-6 and G-CSF were detected 7 days p.i. in unprotected mice. A decrease in both CXCL10 and IL-13 occurred between 7–14 days p.i. in protected mice. B lymphocyte chemoattractant (BLC) CXCL13 was elevated 14 days p.i. in unprotected mice and unaltered in protected mice. Overall, serum cytokines present during infection of unprotected mice are unaltered during the challenge of protected mice. In conclusion, these results indicate that systemic signals for pro-inflammatory cytokines (CXCL1 and IL-6) and recruitment of B-cells (CXCL13), T-cells (CCL11 and CXCL10) and neutrophils (CXCL1, G-CSF) are not required in protected mice during a challenge with *B*. *pertussis*.

### Cellular Composition Spleen

In unprotected mice, neutrophils (Gr1^+^) play an important role in the clearance of bacteria from the lungs and are often used as marker of disease. This process is characterized by a gradually increased percentage of neutrophils in the spleen between 4 and 21 days p.i. [10]. Analysis of the percentage Gr1^+^ cells in the spleen following challenge in protected mice indicated that this recruitment of granulocytes did not occur 14 days p.c. whereas the drastic increase of granulocytes in unprotected mice was again observed (Fig 6B). Together, these results show that recruitment of circulating granulocytes/neutrophils occurs only in unprotected mice after a challenge.

### *B*. *pertussis*-Specific Antibody Responses in Serum and Lung Tissue Extract

**Fig 7:**
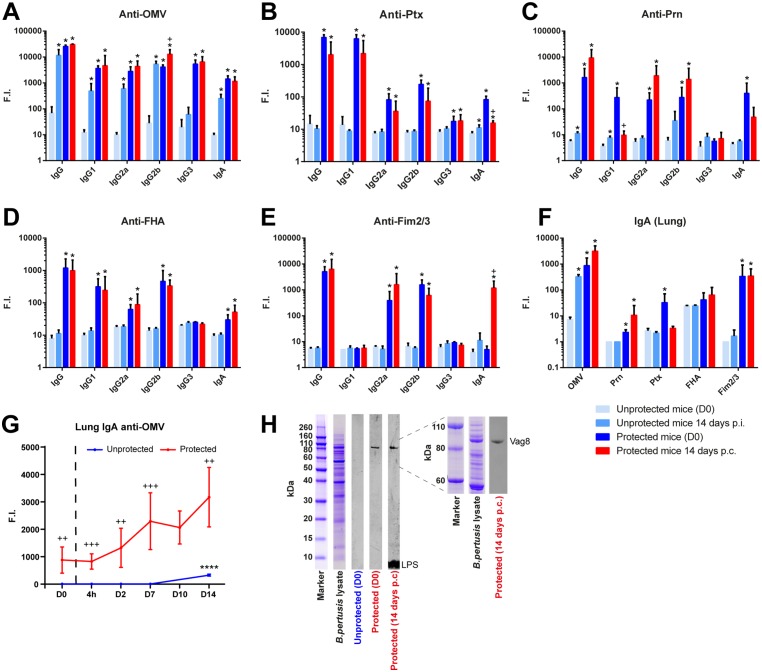
Serum and pulmonary antibody profiles in unprotected and protected mice following *B*. *pertussis* challenge. Serum IgA, IgG, and IgG subclass responses specific for (A) OMV, (B) Ptx, (C) Prn, (D) FHA, and (E) Fim2/3 were determined by using a MIA. Data were obtained in naive mice and protected mice prior to challenge (D0) and 14 days post infection (p.i.) or 14 days post-challenge (p.c.) (n = 3/time point). (F) Pulmonary IgA responses against these antigens were determined on the same time points. * = p<0.05 experimental group versus unprotected group (D0), + = p<0.05 protected group (D0) versus protected group (14 days p.c.). (G) The kinetics of the anti-OMV IgA antibody formation in lung lysates were analyzed at more time points (n = 3/time point) and expressed in fluorescence intensity (F.I.). **** = p<0.0001, challenged unprotected or protected group versus unprotected or protected group (day 0), ++ and +++ = p<0.01 and p<0.001 unprotected group versus protected group (for each time point). (H) Western blot on separated *B*. *pertussis* B1917 proteins was performed with pooled lung lysates (1:50) of unprotected and protected mice prior to challenge (D0), and of protected mice 14 days p.c. with IR800-labeled secondary antibody. Left panel shows whole protein range (260kDa-<10kDa) of *B*. *pertussis* lysate. Right panel shows more detailed separation of the 110-60kDa protein range. Antigen identification for Vag8 and LPS is depicted.

Levels of IgA, IgG, and IgG subclasses were determined just before and after 14 days challenge in sera of unprotected and protected mice (Fig 7A–7E) *B*. *pertussis* outer membrane vesicles (OMV) and purified antigens (Prn, Ptx, FHA, and Fim2/3) were used as coating antigen. Only anti-OMV antibodies were induced in unprotected mice 14 days p.i., while antibodies against purified antigens could not be detected. Antibodies specific for all five purified antigens were observed in protected mice before challenge (D0).

After the challenge, anti-Ptx IgG and IgA levels dropped in protected mice, whereas the anti-OMV and anti-FHA IgG levels remained high. Moreover, the anti-Prn IgG1 and IgA levels decreased, while IgG2a and IgG2b levels increased. Notably, serum IgA levels against Fim2/3 were only detected in protected mice 14 days p.c. In addition to systemic antibody levels, the local formation of IgA antibodies was determined in lung lysates (Fig 7F). Challenge in unprotected mice elicited mainly anti-OMV IgA, which was detected 14 days p.i.

In protected mice, anti-OMV IgA antibodies were present before challenge (D0) and were further increased 14 days p.c. Anti-Prn and anti-Fim2/3 IgA remained elevated, while anti-Ptx IgA decreased. Combined data of all time points showed a gradual increase of pulmonary anti-OMV IgA levels post-challenge in protected mice (Fig 7G). Western blotting and mass spectrometry was used to determine the antigen specificity of the anti-OMV IgA antibodies in protected mice before (D0) and after the challenge (D14) (Fig 7H). The IgA antibodies on D0 were solely directed against Vag8. Notably, challenge of protected mice led to strong anti-LPS IgA antibody formation, whereas the level of anti-Vag8 antibodies remained unaltered (Fig 7H).

### Specific CD4^+^ T-Cell Response in the Spleen

**Fig 8:**
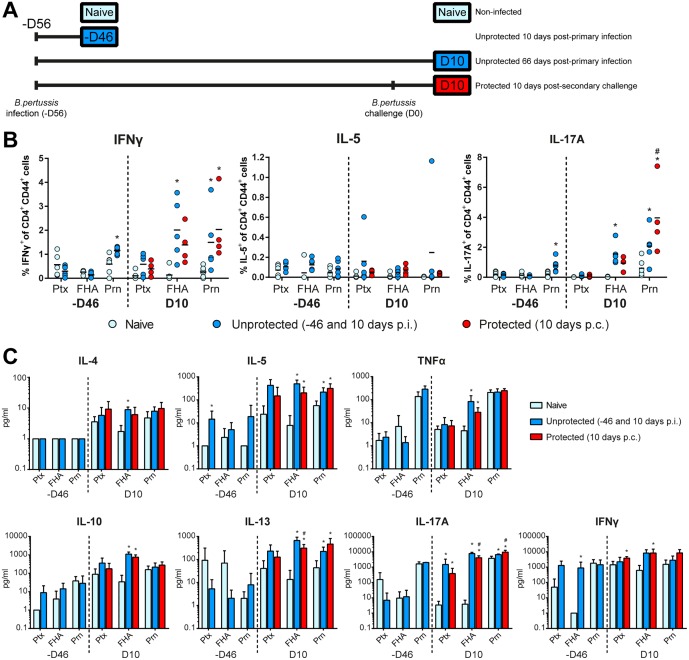
Systemic T-cell responses in unprotected and protected mice. (A) Study design is depicted for the collection of splenocytes in two different experiments (-D46 and D10). The first experiment (-D46) included unprotected mice 10 days p.i. and naive mice. The second experiment (D10) included unprotected mice 66 days p.i., protected mice 10 days post-secondary challenge, and naive mice. Splenocytes were *in vitro* restimulated with Prn, FHA or Ptx for 8 days. (B) The percentages of IFNγ-, IL-5-, and IL-17A-producing CD4^+^CD44^+^ T-cells were determined using ICS (n = 6). (C) Cytokine levels of IL-4, IL-5, IL-10, IL-13, IL-17A, IFNγ, and TNFα in supernatant after 7 days of stimulation were determined by using a MIA. Results (mean of n = 6) are corrected for the background level in the presence of medium as control. Statistical differences between the groups were detected for the ICS with a non-parametric Mann-Whitney test and for the MIA with a Student t-test on the log-transformed data. * = *p*<0.05 experimental group versus naive group, # = *p*<0.05 protected group (D10) versus unprotected group (D10).

After challenge, the presence and type of *B*. *pertussis* antigen-specific memory CD4^+^CD44^+^ T-cells in the spleen were determined. The cytokine production of CD4^+^ T-cells was analyzed according to the study design depicted in Fig 8A on single cell level and in culture supernatants after stimulation of splenocytes with Ptx, FHA, or Prn. The CD4^+^ T-cell response was determined after primary infection of unprotected mice 10 days and 66 days p.i. (-D46 and D10, respectively). Primary infection led to increased percentages of IFNγ- and IL-17A-producing Prn-specific CD4^+^ T-cells on 10 days p.i. (-D46), which percentages significantly increased 66 days p.i. (D10). In addition, IFNγ- and IL-17A-producing FHA-specific CD4^+^CD44^+^ T-cells could be detected on this time point (Fig 8B). Protected mice received an intranasal *B*. *pertussis* challenge 56 days after the primary infection (D0) and showed increased percentages of IFNγ- and IL-17A-producing Prn-specific CD4^+^ CD44^+^ T-cells on 10 days p.c. compared to the percentage in naive mice. Notably, the percentage IL-17A-producing Prn-specific CD4^+^ CD44^+^ T-cells in these challenged protected mice 10 days p.c. was increased compared to the percentage in the unprotected mice on that time point (Fig 8B).

Cytokine levels in culture supernatant revealed that 10 days p.i. (-D46) splenocytes showed a moderate production of IL-5 and IFNγ after stimulation with Ptx and FHA, respectively (Fig 8C). In unprotected mice that were sacrificed 66 days p.i. (D10), Prn-stimulation resulted in enhanced production of IL-5, IL-13, and IL-17A while production of IL-17A was also observed after Ptx stimulation. FHA stimulation caused production of IL-4, IL-5, IL-13, TNFα, IL-17A, and IL-10. Data obtained in protected mice 10 days p.c. revealed the induction of IL-5, IL-13 and IL-17A after Prn-stimulation, increase in IFNγ- and IL-17A after Ptx-stimulation, and production of IL-5, IL-13, IFNγ, TNFα, IL-17A and IL-10 after FHA-stimulation. In conclusion, infection-induced T-cell responses were Th1 and Th17 mediated. Additionally, challenge of protected mice did not result in a dramatic booster of the CD4^+^ T-cell response, since only a higher percentage of IL-17A-producing CD4+CD44+ was observed in protected compared to unprotected mice

## Discussion

A *Bordetella pertussis* infection in mice or baboons causes sterilizing immunity [7, 10]. There are strong indications that the Th1/Th17 responses and mucosal IgA are major contributors to this superior protection [10–12]. Nevertheless, many immunological responses caused upon a *B*. *pertussis* infection are still not discovered. In the current study, protected and unprotected mice were challenged intranasally with *B*. *pertussis* after which innate and adaptive immunological signatures were identified associated with the infection-induced and excellent immunity in mice.

Protected mice had high levels of *B*. *pertussis*-specific serum IgG, mucosal IgA in the lungs and Th1/Th17 cells in the spleen, whereas unprotected mice have not. In addition, pulmonary molecular signatures in the protected mice revealed unique markers of B-cells, T-cells, and immunoglobulins, implying the presence of tissue-resident adaptive immune cells in the lungs. Moreover, molecular signatures of myeloid and mucosal-specific cells, such as mast cells and goblet cells were present. Interestingly, 320 genes were still differentially expressed before the challenge in protected mice as result of the first infection, as compared to unprotected mice before challenge. Since bacteria were cleared from the lungs 28 days after primary infection [10], the question was raised what is the source responsible for this upregulation. Possibly, a change in the cellular composition of the lungs due to residence of new innate and adaptive immune cells result an aberrant gene expression profiles. Importantly, the overall transcriptomics data indicate not only a relatively persistent and broadly altered adaptive pulmonary immune status but also maintained innate changes in protected mice in comparison to their naive counterparts.

Pathogenesis of pertussis in non-vaccinated individuals is characterized by prolonged bacterial presence and severe lung tissue distress and damage [26]. It is hypothesized that immune evasion strategies of *B*. *pertussis* [27] extend residence time. Ideally, pre-existing immunity induced by vaccination should provide fast clearance of pertussis bacteria and long-lasting immunity to avert bacterial transmission [7] and limit respiratory tissue damage. The absence of pro-inflammatory responses and neutrophil circulation may be signs of less severe disease. Protected mice, in contrast to unprotected mice, had reduced systemic serum cytokine secretion, including the pro-inflammatory cytokine IL-6 and neutrophil attractants (CXCL1 and G-CSF), and no induction of neutrophils in the spleens. Additionally, selective innate molecular signatures were absent in the lungs of protected mice following the *B*. *pertussis* challenge. This included genes encoding pro-inflammatory cytokines such as *Il1a*, *Il1b*, *Il6* and *Saa1* as part of the acute phase response. Moreover, enhanced gene expression of the KLR and CLEC-family, was not detected in protected mice. KLR and CLEC genes may harbor similar functions [28], which are specifically found on myeloid cells [29] and play an important role in tissue infiltration of neutrophils following inflammation [30]. This lack of expression in protected mice implies that myeloid cells expressing these membrane markers are not required for the sterilizing immunity. Finally, the enhanced cell proliferation in the lungs of unprotected mice, based on the high expression of Ki-67, was absent in protected mice. Overall, the altered transcriptomes in the lungs of protected mice suggest that these mice are able to control the challenge locally without alarming systemic pro-inflammatory signals.

The acquired immune status of the protected mice enables an alternative solution for fast bacterial clearance with limited systemic recruitment of immune cells from the circulation. Specific markers (IL-5, TNFα and M-CSF) were exclusively present in the protected mice, which may contribute to the sterilizing immunity. Early secretion of IL-5 indicates the presence of type II innate lymphoid cells (ILCs) or antigen specific Th2-cells in the lungs. Both Th2-cells [31] and ILCs [32] can play a role in protection during the innate phase. However, no *B*. *pertussis*-specific Th2-cell formation was detected in the spleen of protected mice. The presence of ILCs is indicated by gene expression of *Thy1* and *Il7r* [33]. Therefore, IL-5 could be produced by type II ILCs. These type II ILCs also promote early antibody responses in the lung [34]. While pulmonary innate immune cells still play a role during the challenge of protected mice, some of these cells signatures (i.e. *Gp2*, *Umod*, *Pglyrp1*, *Clca3*) were also highly active prior to the challenge. The pulmonary expression of *Gp2* and *Umod* was increased in protected mice prior and post-challenge. Glycoprotein 2 (GP2) and uromodulin (Umod) are expressed on microfold cells (M-cells) [35]. M-cells facilitate the uptake of antigens at the mucosal site and the subsequent transportation across the epithelial barrier. GP2 serves as a receptor for mucosal antigen and binds adhesins on Fim^+^ bacteria [36], such as *B*. *pertussis*. Simultaneously, expression of peptidoglycan recognition protein 1 (*Pglyrp1*) was detected, which at the intestinal mucosal site is found in Peyer’s patches co-localized with M-cells [37]. This indicates that the M-cells are either increased in number or more active in protected mice prior and post-challenge. At the pulmonary site, M-cells are commonly found in the Bronchus-Associated Lymphoid Tissue (BALT) [38] gathered with tissue-resident adaptive cells, such as B-cells and T-cells, facilitating a more efficient antigen presentation. This suggests that the local pulmonary immunity induced by a primary *B*. *pertussis* challenge is a result of not only the induction of acquired immunological memory, but also increased innate activity and subsequent interplay between of innate and acquired immune cells in the BALT. Enhanced expression of genes specific for goblet cells (mucus-producing cells), such as chloride channel calcium activated channels (*Clca1*, *Clca3* and *Clca4*) and anterior gradient 2 (*Agr2*) [39] suggests that these cells are more active during the challenge in protected mice. Goblet cells can also orchestrate local immune responses. For instance, IL-17 and CXCL-1 induction in the lungs and leukocyte recruitment are modulated by CLCA3 in mucus cells [40].

Myeloid cells play an important role in clearance of *B*. *pertussis*. For instance, a large neutrophil influx into the lungs occurs during the challenge of unprotected mice [10, 41]. Neutrophils are essential for phagocytosis and may serve as a marker for disease like white blood cell counts for baboons and young infants [42, 43]. Myeloid cells expressing membrane markers of the KLRs, CLECs and the MS4A family [28, 29, 44] were absent in the protected mice after the challenge. However, the enhanced gene expression of the neutrophil-specific *Cd177* and macrophage-specific *Mpeg1* suggests involvement of other subsets of myeloid cells during the challenge in protected mice. The expression of CD177 on neutrophils is enhanced in the presence of G-CSF [45] which was also detected in the lungs of protected mice. These signatures could be related to changes in the pulmonary cell composition as a result of the first infection. For example, opsonization of bacteria by pre-existing specific antibodies and the subsequent phagocytosis by different myeloid cells enables an enhanced clearance of bacteria. In addition, the outgrowth of bacteria is absent in protected mice after challenge. Hence, the total number of phagocytes required for removal of bacteria is expected to be lower. Finally, the infection-induced immunity might result in signatures of airway specific cells, such as was detected for alveolar macrophages. Both GC-CSF and M-CSF play an important role in proliferation and differentiation of alveolar macrophages [46]. In addition, pulmonary secretion of M-CSF was detected exclusively in protected mice 4 hours p.c. This change in lung environment enables formation of specific alveolar macrophages with a very high antigen-presenting capacity [47]. The contribution of alveolar macrophages [48] and mast-cells [49] with respect to immunity against *B*. *pertussis* has previously been demonstrated.

Together these findings demonstrate that the infection-primed immunity resulted in the enhanced activity of pulmonary innate cells, such as alveolar macrophages, M-cells, and ILCs. This may indicate that these innate cells have trained innate immunity [50] that provides an altered pulmonary innate homeostasis [51]. This different status of innate cells may contribute to the local immunity in protected mice. Therefore, pulmonary innate cells are interesting targets for optimizing pertussis vaccination strategies, e.g. by pulmonary vaccination.

Infection-induced immunity comprised of a Th1/Th17-mediated response, as was demonstrated by the systemic analysis of *B*. *pertussis*-specific T-cells in the spleen. After the challenge, these responses were recalled fast in the protected mice. Despite the upregulated pulmonary gene expression of *Cxcl9*, protein secretion of CXCL9 and CXCL10 was not detectable in lung lysates and serum of protected mice, indicating reduced recruitment of CXCR3^+^ T-cells. However, gene expression showed that molecular signatures of T-cells (i.e. *Cd3g*, *Cd4*, *Thy1*) were further enhanced in protected mice post-challenge. This indicates the presence of tissue-resident memory CD4^+^ T-cells. This assumption is emphasized by the enhanced expression of the memory T-cell marker *Il7r* (*Cd127*). Moreover, gene expression of the chemokines *Ccl20* and *Cxcl12* and their corresponding receptors *Cxcr6* and *Ccr6* implied recruitment of different types of T-cells, such as CCR6^+^ Th17-cells [52] and CXCR6^+^ T-cells [53] in the lungs. Lee *et al*. showed that the presence of pulmonary CXCR6^+^ T-cells correlated with local protective immunity against *Mycobacterium tuberculosis* [53]. Furthermore, CCR6 is an important mediator of mucosal immunity [54], indicating a potential role for pulmonary CXCR6^+^ and CCR6^+^ T-cells in immunity against *B*. *pertussis*. Strong *B*. *pertussis*-specific Th17 responses occur during infection in unprotected mice, which remain present for a long period [10]. Moreover, the Resistin like alpha (*Retnla*) was found to stimulate Th17 responses [24]. However, the secretion of IL-17A and expression of *Il17f* in the lungs was absent after the challenge of protected mice. This indicates either that we missed this expression due to a change in expression kinetics or that the effector function of the Th17 cells had changed.

Protected mice had high levels of *B*. *pertussis*-specific IgG in the serum. In addition, protected mice expressed genes encoding for B-cell markers and antibody production in their lungs. This was confirmed by the presence of mucosal IgA in the lungs. We demonstrated that this pulmonary IgA in protected mice is directed against Ptx, a Fim2/3 antigen mixture, and Vag8. Interestingly, the anti-Ptx IgG and IgA antibody levels decreased after the challenge and may have played a role in the neutralization of pertussis toxin during the second infection. The inclusion of Fimbriae or Vag8 in pertussis vaccines may improve the efficacy. Especially targeting Vag8, an important virulence factor that affects complement-mediated responses [55, 56], may significantly improve the efficacy of pertussis vaccines by enhancing opsonophagocytosis [56–58]. IgA plays an important role in protection against *B*. *pertussis* [59]. Therefore, the induction of mucosal IgA by mucosal vaccination might be a big advantage, especially because IgA induction is completely absent after immunization with current pertussis vaccines [58]. Induction of B-cell-related gene expression after a challenge of protected mice shows a recall response leading to increased antibody production. Here, CCR6 expression may be important for memory B-cells to mount a recall response [60]. The antibody-related gene expression showed co-expression with components of the classical complement pathway and seven genes of the SPEER-family with unknown function, suggesting the expression of these genes occurring in the B-cells. Overall, these findings demonstrate the presence of both systemic and pulmonary humoral responses primarily directed against Ptx, Fim2/3 and Vag8. This is where the results obtained in this study may contribute to understanding and optimizing immunity against *B*. *pertussis*. The different pertussis vaccines, such as the acellular (aPV), whole-cell (wPV) and outer membrane vesicle (omvPV) vaccines, evoke substantially different T-helper cell polarization [11, 61] and humoral immunity, based on subclass responses and antigen specificity [58]. All vaccines provide however enhanced bacterial clearance in mice as compared to non-vaccinated mice, indicating that multiple types of immunity can achieve protection against pertussis. Nonetheless, sterilizing immunity in the lungs that is seen for infection induced-immunity [7, 10] is not provided by subcutaneous vaccination [7].

In conclusion, the comprehensive comparison of responses between protected mice and unprotected mice resulted in detailed insight in characteristics of protective immune responses against pertussis. The unprotected mice are largely depending on recruitment of phagocytic cells into the lungs to remove the bacteria, whereas the acquired immunity in the protected mice enables fast recognition and neutralization of the bacteria. The presence of mucosal IgA and upregulation of gene signatures related to memory Th1/Th17 cells and memory B-cells may indicate the presence of these cells that contribute to effective immunity against *B*. *pertussis*. Here, CCR6^+^ B-cells, Th17 cells and CXCR6^+^ T-cells play an important role in the *B*. *pertussis* infection-induced pulmonary immunity. Moreover, high activity of ‘trained’ pulmonary innate immune cells, such as alveolar macrophages, M-cells and goblet cells, was observed in the lungs of protected mice. Hence, induction of pulmonary immunity e.g. achieved by local immunization will improve the efficacy of pertussis vaccines, perhaps also on the long term. A single dose of intranasally administered live-attenuated pertussis vaccine shows that such an approach induces good protection in mice [62] with longer lasting immunity [63] and also through a Th1/Th17-mediated response [64]. In conclusion, the present study showed that the use of a systems approach enables to unravel infection-induced responses in more detail, including the interplay between innate and acquired immune responses.

## Methods

### Ethics Statement

An independent ethical committee for animal experimentations (DEC) of Intravacc reviewed the animal experiments of this study according to the guidelines provided by the Dutch Animal Protection Act. The document of this study with the identification number ‘DPA201100348’ was approved by the committee.

### *B*. *pertussis* Challenge Culture

Cultivation of *Bordetella pertussis* B1917 and preparation of the challenge culture was performed as previously described [10].

### Animal Experiments

Thirty female, 8-weeks-old, BALB/c mice (Harlan, The Netherlands) were divided in ten groups of three animals and housed in cages (macrolon III including filter top). Six groups of naive mice (n = 3 mice/group) were intranasally infected under anesthesia (isoflurane/oxygen), with 2x10^5^ CFU *B*. *pertussis* B1917 in 40μl Verweij medium and left 56 days to recover. 56 days p.i. (D0), five groups of these protected mice with infection-induced immunity and five groups of naive (D0 unprotected) mice (n = 3) of the same age were challenged intranasally as described above for the primary infection and euthanized at 4 hours, and 2, 7, 10 and 14 days after challenge, according to the study design in Fig 1. Additionally, 30 mice were included for two individual experiments analyzing CD4^+^ T-cell responses (Experiment design in Fig 8A). On 10 days p.i. (-D46), six primary-infected and six complete naive mice were euthanized. On day 10 p.c., six mice that had encountered two infectious doses (-D56 and D0), six primary-infected mice (66 days p.i.), and six complete naive mice were euthanized. Mice from all groups were bled under anesthesia (isoflurane/oxygen) by orbital bleeding and euthanized by cervical dislocation. Collection of whole blood, lungs and spleen were performed as described before [10].

### Microarray Analysis

RNA isolation from lung tissue, determination of RNA concentrations, and RNA integrity was executed as described before [10]. Amplification, labeling and hybridization of RNA samples to microarray chips was carried out at the Microarray Department of the University of Amsterdam, The Netherlands, as described previously [65]. Complete raw and normalized microarray data and their MIAME compliant metadata from this publication have been submitted to the GEO database (www.ncbi.nlm.nih.gov/geo) and assigned the identifier GSE75438.

### Data Analysis of Gene Expression

Statistical analysis of gene expression data was done in R according to methods described before [10]. Further functional interpretation, was performed using resources such as DAVID [18], Gene Ontology, KEGG, and BioGPS. Data visualization was done in R as well as GeneMaths XT software (Applied Maths, Sint-Martens-Latem, Belgium).

### Antibody Levels in Serum and Lung Lysate

Levels of pulmonary total IgA and serum total IgA, IgG and IgG subclass (IgG1, IgG2a, IgG2b and IgG3) specific for *B*. *pertussis* antigens pertactin, filamentous hemagglutinin (FHA), pertussis toxin (Ptx), combined fimbria type 2 and 3 antigens (Fim2/3) and outer membrane vesicles B1917 (OMV B1917) were determined using a multiplex immunoassay (MIA) as described previously [10].

### Western Blotting Using IgA from Lung Lysate

Infrared labeling (IR800) of goat-anti-mouse IgA (Southern Biotech), protein separation by SDS-PAGE, and immunoproteomic profiling by Western blot (1:50 dilution) and MS-MS were performed as described previously [58]. For improved separation of the 80kD-110kD range, a 4–12% Bis-Tris protein gel (NuPAGE Novex, 1.0mm 10wells, Invitrogen) was used.

### Splenic Gr-1^+^ Cell Analysis

Determination of the percentage Gr-1^+^ cells in isolated splenocytes was carried out as described before [10].

### Stimulation of *B*. *pertussis*-Specific T-Cell from Spleens

Splenocytes were isolated and stimulated with 1 μg/ml Prn, Ptx or FHA in IMDM complete medium (IMDM medium (Gibco) supplemented with 8% FCS, 100 units penicillin, 100 units streptomycin, 2.92 mg/ml L-glutamine, and 20 μM β-mercaptoethanol (Sigma)), or only medium, as described previously [10]. Supernatants were collected after 7 days for cytokine analysis. After overnight stimulation with the same conditions, intracellular cytokine staining was executed splenocytes that were stimulated for 8 days to identify CD4^+^CD44^+^ T-cells producing IL-5, IFNγ or IL-17A.

### Multiplex Immunoassay (MIA) for Cytokines

A mouse cytokine 7-plex luminex kit (Milliplex; Merck KGaA, Darmstadt, Germany) was used to determine concentrations (pg/ml) of IL-4, IL-5, IL-10, IL-13, IL-17A, TNFα, and IFNγ in culture supernatants of 7 days stimulated splenocytes of individual mice in a MIA. A mouse cytokine/chemokine 32-plex luminex kit (Milliplex MAP Mouse Cytokine/Chemokine—Premixed 32 Plex; Merck KGaA, Darmstadt, Germany) was used to determine concentrations (pg/ml) of Eotaxin, G-CSF, GM-CSF, IFNγ, IL-10, IL-12 (p40), IL-12 (p70), IL-13, IL-15, IL-17A, IL-1α, IL-1β, IL-2, IL-3, IL-4, IL-5, IL-6, IL-7, IL-9, IP-10, KC, LIF, LIX, M-CSF, MCP-1, MIG, MIP-1α, MIP-1β, MIP-2, RANTES, TNF-α, and VEGF, in serum and lung lysates in a MIA (according to the manufacturer’s instructions and as described previously [10]). Measurements and data analysis were performed with Bio-Plex 200, using Bio-Plex Manager software (version 5.0, Bio-Rad Laboratories).

### BLC (CXCL13) Cytokine ELISA

Serum concentration of B lymphocyte chemoattractant (BLC/CXCL13) was determined by ELISA (Mouse BLC ELISA kit, RAB0046, Sigma-Aldrich, Germany) according to the manufacturer’s manual. In short, serum (50 μl) was diluted in assay buffer A (1/1; v/v) and incubated in an antibody-coated ELISA plate (overnight, 4°C, gentle shaking). After washing four times with 300 μl wash buffer, wells were incubated with 100 μl biotinylated antibody (1 hour, room temperature, gentle shaking). After washing four times with 300 μl wash buffer, wells were incubated with 100 μl streptavidin solution (45 min., room temperature, gentle shaking). After washing four times, wells were incubated with 100 μl TMB one-step substrate reagent (30 min., RT) and reaction stopped with 50 μl stop solution. Finally, the absorbance was recorded at 450 nm with a plate reader (BioTek reader EL808, Bio-Tek) and converted to BLC concentration by using a dilution series of a standard (1.37–1,000 pg/ml) and Gentech 5 software (BioTek).

### Statistical Analysis

Serum and pulmonary cytokines levels were analyzed by using a one-way ANOVA with multiple comparisons compared to the pre-challenge level (D0) of unprotected mice or protected mice followed by a Dunnett t-test. ICS data was analyzed by using a Mann-Whitney t-test. For cytokine profiling after re-stimulation of splenocytes, cellular composition of spleen, and antibody levels, data was first log-transformed before t-test analysis. In all corresponding figures, *p*-values are represented as * = *p* <0.05, ** = *p* <0.01, *** = *p* <0.001 and **** = *p* <0.0001.

## Supporting Information

S1 Fig *Comparison of pulmonary transcriptomic profiles between* unprotected and protected mice per time point. (A) The fold ratios (FR) of all 786 differentially regulated genes compared to non-infected naive mice for both unprotected and protected mice were portrayed as scatter plot at all five time points. At each time point, for each gene, the FR was calculated between unprotected and unprotected mice. All genes that were differently expressed (FR ≥ 1.5 or ≤0.67) were depicted in red. The number of upregulated and downregulated genes is given for each comparison. (B) The 212 genes that were found differently expressed (FR ≥ 1.5 or ≤0.67) between unprotected and protected mice 2 days p.c. in Fig 2D were divided in in eight fractions based on upregulation (1–4) or downregulation (5–8). These genes are depicted as heatmap per fraction with additional information on the FR of these genes in unprotected mice and protected mice compared to non-infected naive mice.
(TIF)

S1 Table Differentially expressed genes in the lungs of protected and unprotected mice following a *B*. *pertussis* challenge. Full list of 786 genes that are differentially expressed in the lungs of protected and unprotected mice following a *B*. *pertussis* challenge. Information includes official gene symbol, cluster number, expression profiles, EntrezID, Pubmed link, and full gene name.
(XLSX)
