# Supplementary figures and images for: Immunological Signatures after Bordetella pertussis Infection Demonstrate Importance of Pulmonary Innate Immune Cells
Source: PLoS One. 2016 Oct 6;11(10):e0164027. doi: 10.1371/journal.pone.0164027 (PMC5053408; doi:10.1371/journal.pone.0164027)

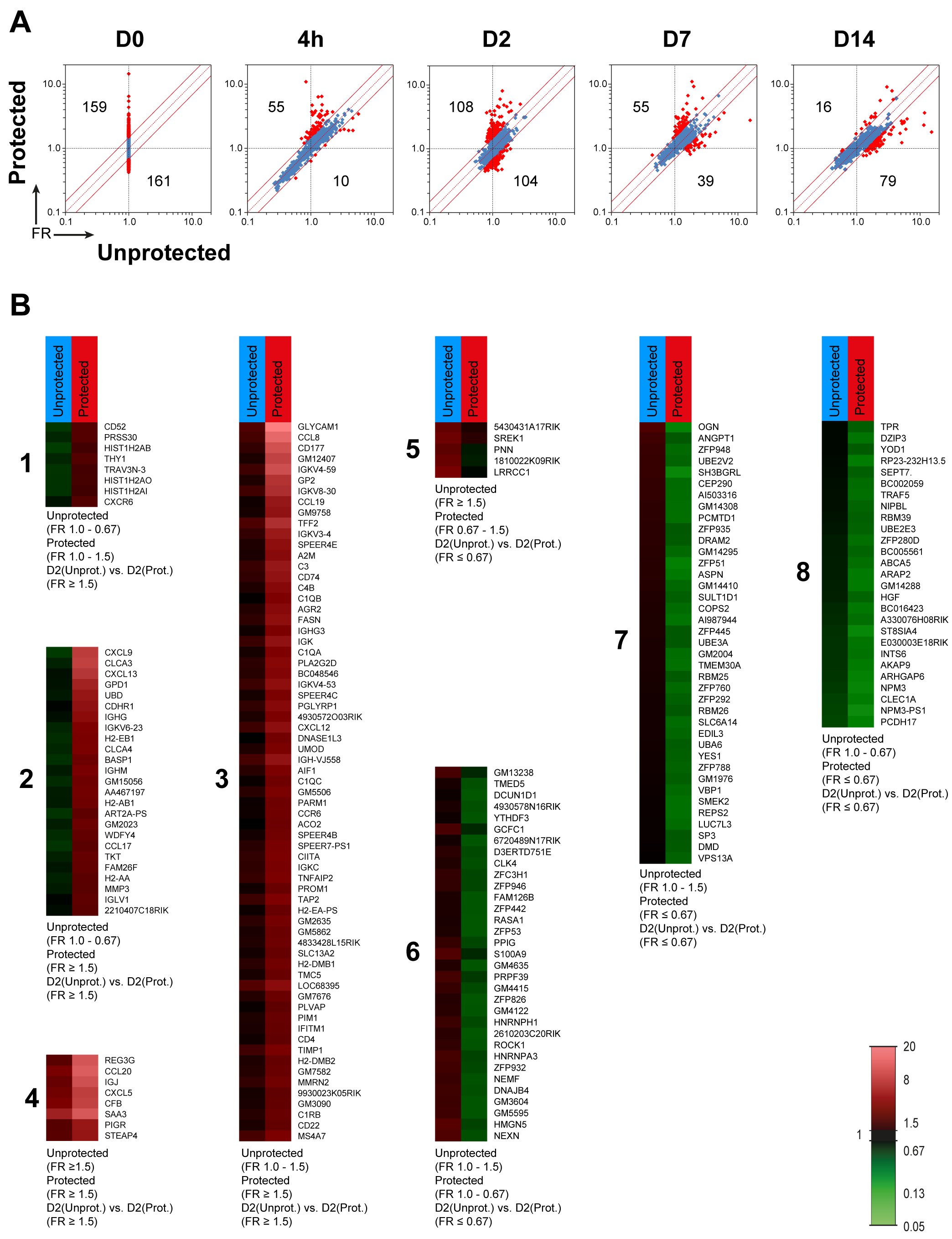

Supplement: S1 Fig — (A) The fold ratios (FR) of all 786 differentially regulated genes compared to non-infected naive mice for both unprotected and protected mice were portrayed as scatter plot at all five time points. At each time point, for each gene, the FR was calculated between unprotected and unprotected mice. All genes that were differently expressed (FR ≥ 1.5 or ≤0.67) were depicted in red. The number of upregulated and downregulated genes is given for each comparison. (B) The 212 genes that were found differently expressed (FR ≥ 1.5 or ≤0.67) between unprotected and protected mice 2 days p.c. in Fig 2D were divided in in eight fractions based on upregulation (1–4) or downregulation (5–8). These genes are depicted as heatmap per fraction with additional information on the FR of these genes in unprotected mice and protected mice compared to non-infected naive mice. (TIF) [file pone.0164027.s001.tif]
